# A cohort study of intrapartum group B streptococcus prophylaxis on atopic dermatitis in 2-year-old children

**DOI:** 10.1186/s12887-022-03758-5

**Published:** 2022-12-02

**Authors:** Zhang Hong, Ren Jing, Li Hui, Xu Kang, Zhang Chunmei, Wang Yang, Zhou Baojian, Ding Xin, Yin Xiaoping

**Affiliations:** 1 Taixing People’s Hospital, Taizhou, Jiangsu China; 2 Children’s Hospital of Soochow University, Soochow, Jiangsu China

**Keywords:** Group B streptococcus, Intrapartum antibiotic prophylaxis, Atopic dermatitis

## Abstract

**Objective:**

To understand the occurrence of atopic dermatitis (AD) in children aged 2 years on exposure to maternal group B streptococcus (GBS) antibiotic prophylaxis (IAP).

**Design:**

Retrospective cohort study of 2909 mother–child pairs.

**Setting:**

Taixing People’s Hospital in Eastern China.

**Participants:**

Term infants born 2018–2019, followed longitudinally from birth to 2 years.

**Exposures:**

The GBS-IAP was defined as therapy with intravenous penicillin G or ampicillin or cefazolin ≥ 4 h prior to delivery to the mother. Reference infants were defined as born without or with other intrapartum antibiotic exposure.

**Outcomes:**

The logistic regression models were employed to analyze the effect of intrapartum GBS prophylaxis on AD in 2-year-old children during delivery. Analysis was a priori stratified according to the mode of delivery and adjusted for relevant covariates.

**Results:**

The cohorts showed that preventive GBS-IAP was potentially associated with increased incidence of AD in children delivered vaginally according to logistic regression models before and after covariate-adjusted treatment (OR: 6.719,95% CI: 4.730–9.544,*P* < 0.001;aOR: 6.562,95% CI: 4.302–10.008, *P* < 0.001).

**Conclusion:**

Prophylactic treatment of intrapartum GBS may raise the risk of AD in vaginally delivered children. These findings highlight the need to better understand the risk between childhood AD and current GBS-IAP intervention strategies.

**Supplementary Information:**

The online version contains supplementary material available at 10.1186/s12887-022-03758-5.

## Highlights

► Maternal group B streptococcus antibiotic prophylaxis (GBS-IAP) was associated with the occurrence of atopic dermatitis in 2-year-old children with vaginal delivery.

► The findings were significant in vaginal delivery before and after adjusting for multiple covariates.

**Supplementary Information:**

The online version contains supplementary material available at 10.1186/s12887-022-03758-5.

## Introduction

Research over the past decades has revealed the emergence of group B Streptococcus (GBS) is the leading cause of neonatal infections in China [1]. Intrapartum antibiotic prophylaxis (IAP) was initially introduced in the 1980s to lower the prevalence of perinatal GBS disease and offered to women with particular obstetric risk factors during parturition [2, 3]. The treatment of IAP has significantly diminished the incidence of neonatal GBS early-onset disease (GBS-EOD) in newborns [1, 4]. However, new research has shown that using maternal antibiotics during pregnancy increases the incidence of atopic dermatitis in offspring [5].

Atopic dermatitis (AD), a chronic and recurrent inflammatory skin disease that commonly occurs in infancy, is clinically characterized by intense itching and eczematous lesions, and its incidence has increased sharply over the past decade in response to lifestyle changes [6]. A multicentric study conducted between January and December 2014 showed that the overall point prevalence of AD in infants reached 30.48% in China [7]. Notably, previous studies evidenced the associations between the occurrence of allergic diseases and gut microbiota after cesarean section in children [8]. Recent reports also evidenced the shifts of neonatal gut microbiota when intrapartum antibiotics are administered [9, 10]. However, whether such antibiotic-mediated effects are related to childhood AD is unknown.

To better understand the association between GBS-IAP and AD in children, a retrospective cohort study was administrated in the present project by analyzing 2909 full-term newborns approximately 2 years old to establish a theoretical basis for formulating GBS intervention strategies in China.

## Methods

### Ethics approval and consent to participate

The study was approved by the Ethics Committees of Taixing People’s Hospital (Reference txry2018-003) in Jiangsu Province, China. Written informed consents were obtained from the parents of all the enrolled infants before data collection. All methods were carried out in accordance with relevant guidelines and regulations.

### Design and setting

The subjects selected pregnant women and full-term newborns delivered in the present hospital from June 2018 to December 2019. A total of 3500 pregnant women were enrolled in the study. Among them, 320 were excluded from miscarriage and induced labor, 271 for incomplete information on mother–child pairs, and incomplete disease records. Finally, 2909 pairs of mother–child that met the criteria were included and divided into two cohorts, *i.e. *vaginal delivery, and cesarean section according to the mode of delivery.

### Population

**Fig. 1:**
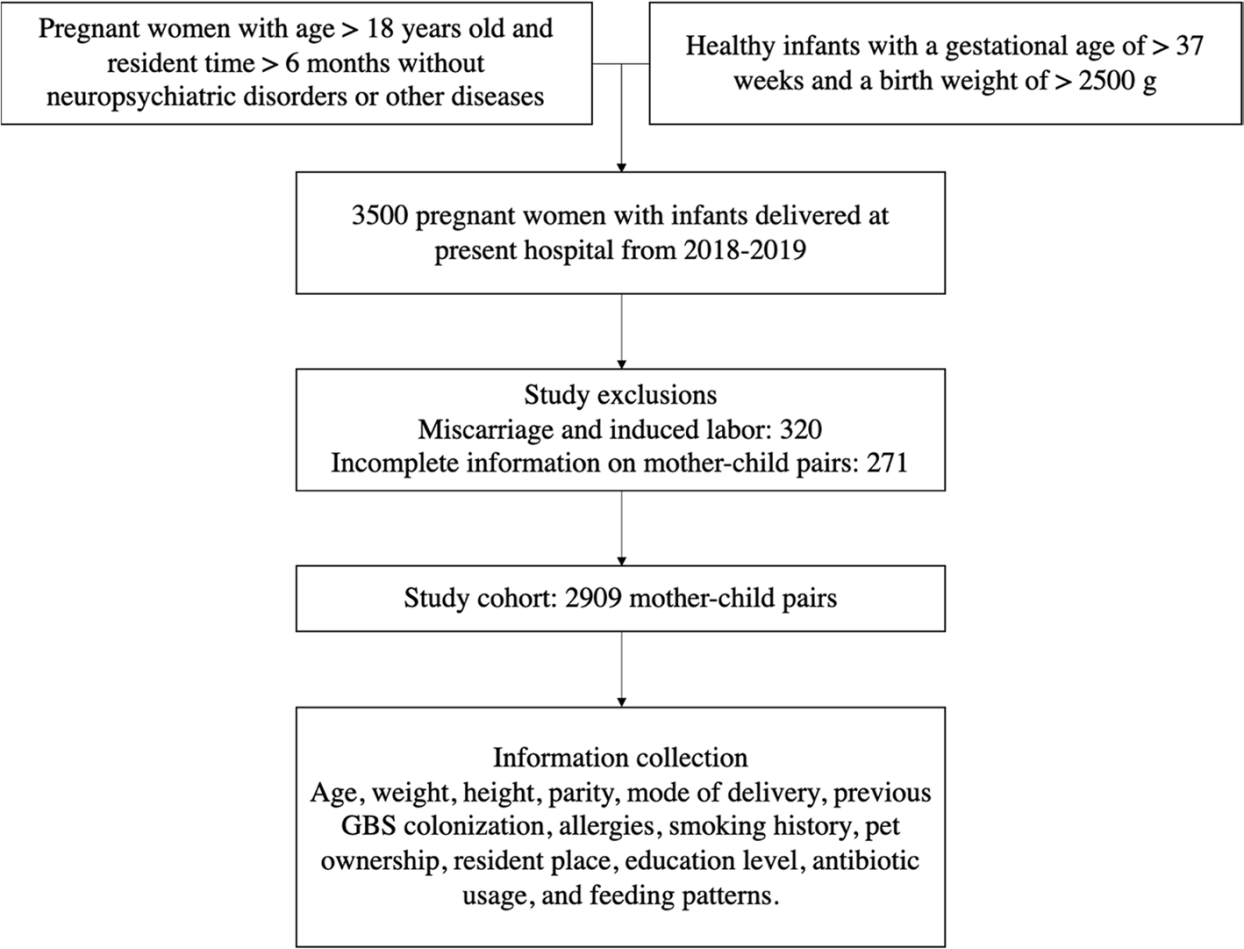
Design of study cohorts. The figure describes the process of inclusion and exclusion criteria for the selected pregnant women and infants

Healthy infants with a gestational age of > 37 weeks and a birth weight of > 2500 g were included in the study. The following criteria that may impact the exposure or outcome were excluded for the subjects (Fig. 1).

### Data collection

A questionnaire was used to collect basic information on the pregnant women and newborns, including the data of age, weight, height, parity, mode of delivery, previous GBS colonization, allergies, smoking history, pet ownership, resident place, education level, antibiotic usage, and feeding patterns. A hospital record system was applicated to collect the data on GBS colonization and IAP intervention for pregnant mothers. GBS colonization was determined according to prenatal GBS-PCR.

### Exposure

The intrapartum antibiotic prophylaxis (IAP) was defined that pregnant women with positive GBS screening were given antibiotic prophylaxis, by intravenous injection of penicillin G or ampicillin or cefazolin at an interval of ≥ 4 h prior to the labor delivery [11, 12]. All the pregnant women with positive GBS screening in this study received the IAP as recommended by national guidelines during the study period. All other forms of maternal antibiotic therapy were classified as “no GBS-IAP” antibiotic exposure, including non GBS-specific antibiotics, GBS-specific antibiotics administered < 4 h before delivery, and surgical prophylaxis administered to women undergoing cesarean delivery. The definition of exposure was applicated identically for both delivery cohorts. Notably, there was a number of 2.5% and 5.1% population from the vaginal birth C-S group were not received IAP treatment due to the refusion of the family member or other urgent issues. Besides, IAP was also given to those pregnant women with GBS positive on their first parity, although the current screen test was negative. Detail exposure issues were listed in Table 1.

**Table 1:** Basic characteristics of the two groups of cohorts

Basic situation	Vaginal birth (*n* = 1510)				Cesarean section (*n* = 1399)			
	No IAP (*n* = 1346)	GBS-IAP (*n* = 164)	*t/χ 2*	*P*	No IAP (*n* = 1279)	GBS-IAP (*n* = 120)	*t/χ 2*	*P*
Mother’s age (years)	27.3 ± 3.9	27.5 ± 4.1	-0.379	0.414	29.0 ± 4.4	29.2 ± 4.5	-0.382	0.702
Mother’s BMI (x ± s)	20.8 ± 1.6	21.0 ± 1.5	-1.094	0.274	21.0 ± 1.8	21.0 ± 1.9	-0.272	0.786
Parity (n, %)			5.733	0.017			2.521	0.112
One child	724 (53.8)	72 (43.9)			596 (46.5)	65 (54.1)		
Second child or more	622 (46.2)	92 (56.0)			683 (53.4)	55 (45.8)		
GBS Screening (n, %)			1206.195	< 0.001			730.014	< 0.001
Positive	33 (2.5)	162 (98.7)			65 (5.1)	108 (90.0)		
Negative	1313 (97.5)	2 (1.3)			1214 (94.9)	12 (10.0)		
Mother’s allergy history (n, %)	128 (9.5)	12 (7.3)	0.835	0.361	110 (8.6)	14 (11.7)	1.277	0.258
Whether received higher education (n, %)	1081 (80.3)	132 (80.5)	0.003	0.957	922 (72.1)	91 (75.8)	0.770	0.380
Gender (man, n, %)	702 (52.1)	103 (62.8)	6.662	0.010	711 (55.6)	58 (48.3)	2.334	0.127
Gestational age (weeks x ± s)	39.5 ± 1.0	39.2 ± 1.1	1.645	0.100	39.3 ± 1.0	39.4 ± 1.0	-1.210	0.227
Weight of birth (g x ± s)	3362 ± 396	3416 ± 415	-1.644	0.100	3462 ± 447	3542 ± 473	-1.850	0.065
Feeding method (n, %)			10.846	0.004			1.336	0.113
Breast feeding	1060 (78.7)	138 (84.1)			963 (75.2)	96 (80.0)		
Artificial feeding	12 (8.9)	5 (3.0)			24 (1.8)	2 (1.7)		
Mixed feeding	274 (20.4)	21 (12.8)			292 (22.8)	22 (18.3)		
Whether used antibiotics in infant (n, %)	92 (6.8)	68 (41.4)	185.044	< 0.001	99 (7.7)	46 (38.3)	110.521	< 0.001
Incidence of atopic dermatitis in 2 years children (n, %)	150 (11.1)	75 (45.0)	140.204	< 0.001	163 (12.7)	9 (7.5)	3.370	0.185

*BMI* Body mass index, *GBS* Group B Streptococcus, *IAP* Intrapartum antibiotic prophylaxis

### Outcome

The primary outcome was the occurrence of AD in infants. In this study, a questionnaire was enrolled to collect AD information from family members including the key question "Had the respondent ever been diagnosed with AD by a doctor or specialist?". The diagnostic of AD in infants was obtained by trained clinical staff according to Williams standards, and an infant was confirmed to have AD when > 3 criteria were present. Detailed criteria were listed in Supply Table 1.

### Covariates

In addition to the exposure factors and mode of delivery, potential confounding factors that may affect outcomes were screened including genetic and environmental issues [1, 11–14]. A questionnaire was used to collect the covariates information of pregnant women and newborns including age, weight, height, parity, mode of delivery, previous GBS colonization, allergies, smoking history, pet ownership, resident place, education level, antibiotic usage, and feeding patterns. The data sources and definitions of model covariates were listed in Supply Table 1.

### Statistical analysis

Initially, chi-square tests and one-way ANOVA were employed for comparison between groups of two cohorts. Then a univariate logistic regression model was performed to analyze the exposure and preselected risk factors for atopic dermatitis in children. To better evaluate the association between maternal IAP and AD in children, a multivariate logistic regression model was enrolled to adjust the confounding factors such as maternal age, maternal allergy history, parity, gestational age, neonatal birth weight, etc. The Data were imported into Epidata 3.0 database. SPSS 22.0 software was used for statistical analysis. The forest plots were drawn with R 4.1.3. Two-sided tests were employed with an inspection level set at α = 0.05.

## Results

### Overview of the cohorts

A total of 2909 mother–child pairs were included in the cohort study (Table 1). Of these, 1510 cases (52%) were delivered through the vagina and 1399 cases (48%) were by cesarean section. Furthermore, 368 pregnant mothers were screened positive for GBS with a colonization rate of 12.6%, with 284 (9.8%) receiving GBS-IAP, comprising 164 cases from the vaginal group and 120 cases from the cesarean section group.

### Characteristics of the cohorts

Multiple characteristics of mother–child pairs were compared between the IAP and non-IAP groups in the two cohorts. As shown in Table 1, characteristics were detected across the vaginal birth cohorts in terms of breastfeeding rates, and antibiotics usage within 72 h after birth (Table 1). In the cohort of vaginal delivery, the IAP group had a higher rate of multipara compared with the control group. Moreover, the prenatal IAP group also had higher GBS-positive rate, frequent maternal breastfeeding, and higher antibiotic use within 72 h of birth in the vaginal delivery cohort (Table 1). In the cohort of cesarean section, significances were observed in the IAP group on primipara, GBS-positive rate, maternal breastfeeding and children were more likely to receive antibiotics use within 72 h after birth in this cohort (Table 1).

### Outcome of the cohorts

Atopic dermatitis affected 397 (13.6%) of the children in the present cohorts, of which 225 (56.7%) were delivered vaginally and 172 (43.3%) were delivered by cesarean section (Table 1). To be specific, the GBS-IAP group had a higher incidence of atopic dermatitis (45% vs 11.1%) compared with the control group in the vaginal delivery cohort (*p* < 0.01). However, in cesarean delivery, the presence of AD was not associated with intrapartum GBS-IAP exposure (7.5% vs. 12.7%, *p* > 0.05).

### Analysis of risk factors for atopic dermatitis

A univariate logistic regression model was initially performed to analyze the exposure and preselected risk factors for atopic dermatitis in children. Results showed that children exposed to GBS-IAP had an increased incidence of atopic dermatitis in the vaginal delivery cohort compared to those without GBS-IAP exposure (OR: 6.719, 95% CI: 4.730–9.544, Table 2). Particularly, the vaginal delivery cohort also revealed an association between childhood AD and other factors such as maternal allergy history (OR: 2.357, 95% CI: 1.575–3.527), mixed feeding rate (OR: 1.479, 95% CI: 1.061–2.061), parity (OR: 1.509, 95% CI: 1.134–2.007), and antibiotic usage (OR: 2.544, 95% CI: 1.793–3.609, Table 2). In the cohort of cesarean section, however, only maternal allergy history (OR: 1.721,95% CI: 1.061–2.792) was evidenced to be associated with the incidence of atopic dermatitis while no significance was observed between the GBS-IAP group and control group in 2 years children (OR: 0.555,95% CI: 0.276–1.117, Table 2).

**Table 2:** Univariate logistic regression analysis of variables in the cohorts

Variables	Vaginal delivery			Cesarean section		
	OR	95%CI	*P*	OR	95%CI	*P*
No IAP	Reference			Reference		
GBS-IAP	6.719	4.730–9.544	** < 0.001**	0.555	0.276–1.117	**0.099**
Mothers age	1.018	0.982–1.056	0.322	0.997	0.962–1.034	0.873
Mothers without allergic history	Reference			Reference		
Mothers with allergic history	2.357	1.575–3.527	< 0.001	1.721	1.061–2.792	0.028
One child	Reference			Reference		
Second child and more	1.509	1.134–2.007	0.005	0.906	0.658–1.246	0.543
Gestational age	0.696	0.607–0.798	< 0.001	1.135	0.965–1.335	0.127
BMI	0.976	0.894–1.065	0.584	0.964	0.881–1.054	0.421
Weight of birth	1.238	0.87–1.762	0.236	1.159	0.816–1.646	0.411
Breast feeding	Reference			Reference		
Artificial feeding	0.386	0.051–2.929	0.357	2.283	0.899–5.795	0.082
Mixed feeding	1.479	1.061–2.061	0.021	1.207	0.832–1.753	0.321
No antibiotics used within 72 h				Reference		
Antibiotics used within 72 h	2.544	1.793–3.609	< 0.01	0.741	0.417–1.318	0.308
No smoking	Reference			Reference		
Smoking	1.420	0.933–2.161	0.102	1.012	0.593–1.728	0.966
No keeping pets	Reference			Reference		
Keeping pets	1.140	0.849–1.532	0.384	1.329	0.950–1.859	0.097

*P* value is for the comparison of the GBS IAP groups among vaginal and caesarean cohorts

*OR* Odds ratio, *BMI* Body mass index, *GBS* Group B Streptococcus, *IAP* Intrapartum antibiotic prophylaxis

In addition, we combined the cohorts and analyzed the total population as a whole group. Results showed that the key variable of GBS-IAP still influenced the occurrence of AD in infants (aOR: 2.953, 95% CI: 2,154–4.048, *P* < 0.001, Table 3). Notably, the vaginal delivery (aOR: 1.000) had a higher effect on AD compared with cesarean section (aOR: 0.794, *p* = 0.045) among the whole population without enrolling IAP as the factor. Then we compared the infant’s AD in those no GBS-IAP received populations according to the modes of delivery. After removing the GBS-IAP factor from the overall population cohort, there were no significant variances in the outcome of AD between the cohorts of vaginal delivery and cesarean section (Tables 4 and 5).

**Table 3:** Multivariate logistic regression analysis of variables in the total 2909 mother–child pairs. The effect of GBS-IAP on AD outcomes remained after the vaginal and cesarean cohorts were combined

Variables	B	Wald	*P*	aOR	95%CI	
Mother’s age	-0.006	0.188	0.664	0.994	0.965	1.023
Breast feeding		7.937	0.019	1.000		
Artificial feeding	0.117	0.073	0.787	1.124	0.483	2.613
Mixed feeding	0.371	7.936	0.005	1.449	1.120	1.876
Weight of birth	0.217	2.749	0.097	1.243	0.961	1.607
Mothers without allergic history				1.000		
Mothers with allergic history	0.690	17.949	**0.000**	1.993	1.449	2.743
No keeping pets				1.000		
Keeping pets	0.217	3.296	0.069	1.242	0.983	1.569
One child				1.000		
Second child and more	0.190	2.316	0.128	1.209	0.947	1.545
Vaginal delivery				**1.000**		
Cesarean section	-0.231	4.023	**0.045**	0.794	0.634	0.995
No IAP				**1.000**		
GBS-IAP	1.083	45.273	**0.000**	2.953	2.154	4.048
Gestational age	-0.128	5.125	0.024	0.880	0.788	0.983
BMI	-0.032	0.952	0.329	0.969	0.909	1.033
No smoking				1.000		
Smoking	0.260	2.082	0.149	1.296	0.911	1.844
No antibiotics used within 72 h				1.000		
Antibiotics used within 72 h	0.115	0.468	0.494	1.121	0.808	1.557

See Supplementary Table 1 for definition of characteristic variable

*aOR* Adjusted odds ratio, *BMI* Body mass index, *GBS* Group B Streptococcus, *IAP* Intrapartum antibiotic prophylaxis

**Table 4:** Comparison of AD in the no GBS-IAP received population by different modes of delivery

Outcome	Mode of delivery		Statistical analysis	
	Vaginal birth (*n* = 1346)	Cesarean section (*n* = 1279)	*χ 2*	*P*
Without atopic dermatitis	1196	1116	1.599	0.206
With atopic dermatitis	150	163

**Table 5:** Comparison of AD in the no GBS-IAP received population. After removing the GBS-IAP factor from the overall population cohort, cesarean section had no effect on the outcome of AD compared with vaginal delivery

Variables	B	Wald	*P*	aOR	95%CI	
Mother’s age	0.011	0.421	0.516	1.011	0.978	1.044
Breast feeding		8.826	0.012			
Artificial feeding	0.540	1.534	0.215	1.717	0.730	4.036
Mixed feeding	0.392	7.898	0.005	1.480	1.126	1.945
Weight of birth	0.203	1.963	0.161	1.225	0.922	1.626
Mothers without allergic history						
Mothers with allergic history	0.720	16.738	0.000	2.055	1.455	2.901
No keeping pets						
Keeping pets	0.268	4.348	0.037	1.308	1.016	1.683
One child						
Second child and more	0.053	0.144	0.705	1.055	0.800	1.390
Vaginal delivery						
Cesarean section	0.131	1.074	0.300	1.140	0.890	1.460
Gestational age	0.020	0.104	0.747	1.021	0.901	1.156
BMI	-0.055	2.299	0.129	0.947	0.882	1.016
No smoking						
Smoking	-0.237	0.978	0.323	0.789	0.493	1.262
No antibiotics used within 72 h						
Antibiotics used within 72 h	0.552	8.154	0.004	1.737	1.189	2.538

See Supplementary Table 1 for definition of characteristic variable

*aOR* Adjusted odds ratio, *BMI* Body mass index

### Association between the risk factors and atopic dermatitis after adjustment

**Fig. 2:**
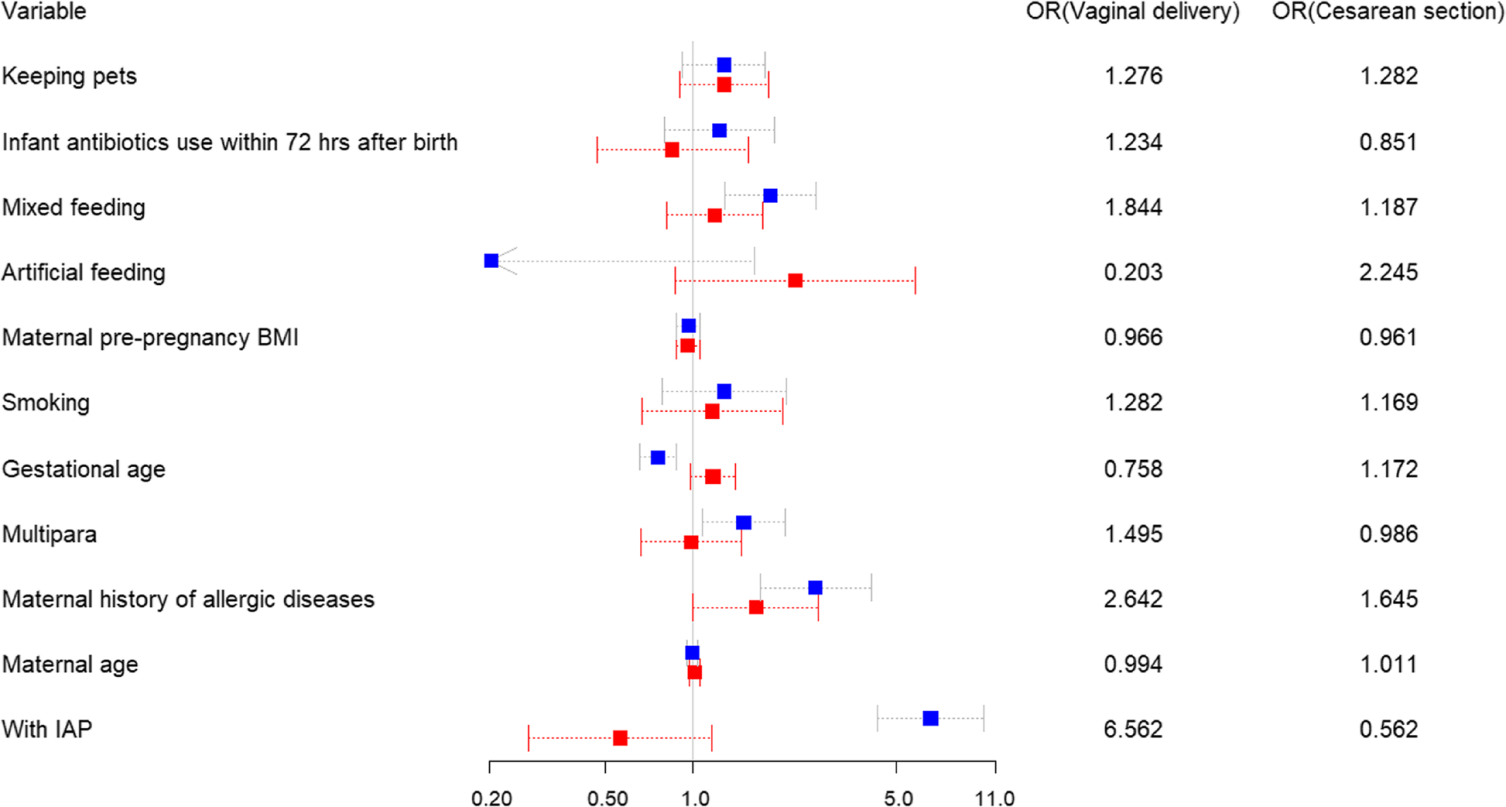
Forest plot for multivariate logistic regression analysis of outcomes. Blue square represents vaginal delivery and red symbol represents cesarean section. See Supplementary Table 1 for definition of characteristic variable. OR, odds ratio

A multivariate logistic regression model was enrolled to explore the association between the cohorts and atopic dermatitis after covariates adjustment. The adjusted results revealed that the vaginal delivery cohort still had a higher occurrence of atopic dermatitis in children with GBS-IAP exposure (adjusted OR: 6. 562, 95% CI: 4.302–10.008, Table 6, Fig. 2). On the contrary, no significant risk factors were identified for atopic dermatitis in children without GBS-IAP exposure delivered by cesarean section (adjusted OR: 0.560, 95% CI: 0.271–1.155, Table 6, Fig. 2). In addition, the vaginal delivery cohort also witnessed the association between childhood AD and other factors in the multivariate logistic regression model including maternal allergy history (adjusted OR: 2.642, 95% CI: 1.699–4.109), mixed feeding rate (adjusted OR: 1.844, 95% CI: 1.281–2.655), and gestational age (adjusted OR: 0.758, 95% CI: 1.003–2.701) that relate to childhood AD. However, only maternal allergy history was evidenced to be associated with the occurrence of childhood AD in the cesarean section cohort (adjusted OR: 1.646, 95% CI: 1.003–2.701, Table 6, Fig. 2).

**Table 6:** Multivariate logistic regression analysis of variables in the cohorts

Variables	Vaginal delivery			Cesarean section		
	aOR	95%CI	*P*	aOR	95%CI	*P*
No IAP	Reference			Reference		
GBS-IAP	6.562	4.302–10.008	** < 0.001**	0.562	0.271–1.155	**0.116**
Mothers age	0.994	0.953–1.036	0.769	1.011	0.969–1.055	0.614
Mothers without allergic history	Reference			Reference		
Mothers with allergic history	2.642	1.699–4.109	0.000	1.645	1.003–2.701	0.049
One child	Reference			Reference		
Second child and more	1.495	1.074–2.081	0.017	0.986	0.664–1.472	0.954
Gestational age	0.758	0.653–0.879	0.000	1.172	0.977–1.4	0.087
No smoking	Reference			Reference		
Smoking	1.282	0.782–2.101	0.324	1.169	0.675–2.057	0.564
BMI	0.966	0.879–1.061	0.470	0.961	0.877–1.053	0.397
Breast feeding	Reference			Reference		
Artificial feeding	0.203	0.025–1.628	0.133	2.245	0.865–5.78	0.097
Mixed feeding	1.844	1.281–2.655	0.001	1.187	0.809–1.734	0.384
No antibiotics used within 72 h	Reference			Reference		
antibiotics used within 72 h	1.234	0.795–1.915	0.348	0.851	0.472–1.564	0.619
No keeping pets	Reference			Reference		
Keeping pets	1.276	0.92–1.77	0.144	1.282	0.905–1.831	0.161

See Supplementary Table 1 for definition of characteristic variable

*aOR* Adjusted odds ratio, *BMI* Body mass index, *GBS* Group B Streptococcus, *IAP* Intrapartum antibiotic prophylaxis

## Discussion

Previous studies have shown an association between postnatal antibiotic exposure and allergic disease in children, while fewer cases of atopic dermatitis were of concern in the context of presumed prenatal antibiotic use. Although there have been previous reports evaluating the association between antibiotic administration during pregnancy and atopic dermatitis in children, the association remains controversial [15–17]. A European cohort report conducted in 2019 revealed a positive association between antenatal antibiotic use and the appearance of AD in the first year of rural-born children [18]. Simultaneously, a prospective birth cohort in China also demonstrated that maternal antibiotic exposure was significantly associated with AD in postnatal childhood [5]. However, a recent meta-analysis claimed that no significant evidence that maternal antibiotic exposure in late pregnancy increases the risk of AD in infants [19].

To better explore the relationship between antibiotic exposure during pregnancy and the risk of atopic dermatitis in children, a retrospective cohort analysis was administrated in this study that identified that vaginal delivery significantly increased the AD risk in children exposed to GBS-IAP. Previous research reported that delivery mode had a greater impact on the establishment of neonatal gut microbiota after birth [13, 20]. Microbiota development was affected in infants born vaginally exposed to IAP for up to 6 months [21]. Specifically, exposure to IAP may change the microenvironment of colonized gut microbiota, leading to a reduction in commensal bacteria but the persistence of pathogenic microorganisms [22]. In the present study, we assumed that the GBS-IAP exposure might result in the disruption of gut microbiota and further influence the course of allergic diseases such as atopic dermatitis processes in childhood. GBS-IAP interventions are usually administered 4 h before delivery, which overlaps with the establishment of gut microbes in the first colonized neonate [17]. Such overlaps may affect the dysregulation of the gut microbiota throughout the neonatal period, and into infancy [23, 24].

Contrary to the cohort of vaginal delivery, no significant incidence of AD was observed in infants with IAP exposure at the time of cesarean delivery. Similar results were observed in previous studies which examined the associations between cesarean delivery and AD. Renz-Polster et al. reported that infants born via C-section without premature rupture of the membranes exhibit a reduction of AD compared with vaginal delivery [25]. Similarly, Kim et al. witnessed a decreased risk of AD among children born via planned C-section [26]. In addition, Rechard et al. demonstrated that the cesarean section was not associated with atopic dermatitis in 4-year-old children in a large cohort in the USA [27]. Such C-section delivery without exposure to maternal microbiome showed no relation to infants’ AD, and was neither affected by intrapartum antibiotics, breastfeeding, missing data, familial factors nor other indications [27]. Furthermore, another study also showed that delivery by cesarean section may cause food allergy in children, but not atopic dermatitis [28], which explained why the colonization of infant gut microbiome was less affected by cesarean section than vaginally born children.

In our study, we collected information on early postnatal antibiotic prophylaxis and postpartum feeding patterns. Our results showed that no significance of risk factors was observed in cesarean mothers compared with the vaginal delivery cohort, consistent with previous studies. This may be explained by the role of gut microbiota perturbation in the dysregulation of immune response and related allergic disorders [29, 30]. Specifically, infants born by vaginal delivery established the microbial communities that resemble maternal vaginal microbiota, whereas CS-delivered newborns obtained those non-maternal bacteria from the hospital environment [31]. Such delayed colonization of beneficial bacteria via CS delivery appeared to be the risk factors that predisposed infants to allergic manifestations. In addition, cesarean section may also affect maturation of immune system by altering stress levels at birth [29]. In contrast to the release of cortisol in fetal circulation with uterine contractions and fetal hypoxia during vaginal delivery, neonates delivered by CS lack the compounds for the maturation of the immune system [29]. This delayed immune maturation may contribute to the occurrence of AD. However, atopic dermatitis is the heterogeneous disorder that involved complex immune responses, environmental exposures, and barrier defects. We agreed with the previous study that an accurate association of CS on AD is unlikely to be revealed by alterations in the gut microbiota in observational studies like ours [28]. Further research is required to illustrate the risk factors of AD by CS delivery.

However, among the studies that evaluated the association between antibiotic administration during pregnancy and allergic disease in infants, Lee et al. and Timm et al. reported a significant connection between prenatal antibiotic exposure and AD in Korean and Danish cohorts [32, 33]. Those children born by cesarean section showed a superior impact on the occurrence of AD. This may be explained by the fact that maternal diseases such as asthma were one of the risk factors for atopic dermatitis, and mothers with asthma were recommended for cesarean section [32]. In this study, only a small proportion (7.3% to 11.7%) of the enrolled pregnant women were reported to have allergy history. After excluding maternal disease bias, we adjusted for AD-related variables and not observed a significant association between the infants’ AD in cesarean mothers who received GBS-IAP.

The antimicrobials administered in this study were narrow-spectrum antibiotics, which may lead to changes in the composition and diversity of gut microbiota during the perinatal period. After therapy with the narrow-spectrum penicillin, Nogacka and Aloisio found a decrease in symbiotic bacteria but an increase in pathogen abundance in infants [34, 35]. Taipianen revealed that the effect size of IAP was comparable to that caused by postnatal antibiotics in newborn infants [36]. When narrow-spectrum antibiotics were given to pregnant women, the effects on the newborn gut microbiota were comparable to when broad-spectrum antibiotics were given directly to neonates. Moreover, Cox reported that postnatal administration of low-dose penicillin induces metabolic changes and affects the ileal expression of immune-related genes [37].

Other genetic and environmental factors, such as the history of family allergic disease, climate change, passive smoking, pet ownership, and other confounding factors, were believed to be connected with the incidence of atopic dermatitis [38]. To better adjust the logistic regression model, these indicators, initially screened by univariate logistic regression, were selected for multivariate regression analysis. The adjusted results showed that maternal history of allergic disease and IAP exposure was an independent risk factor. Parental allergic disease may affect skin barrier function and immune homeostasis, leading to a variety of immunological abnormalities [39]. Other studies have reported that pet ownership and smoking may be linked with the development of atopic dermatitis in children, however, this was not found in our study [40–42].

There are some limitations to this study. First, this study was a retrospective analysis but relied on parental feedback on a diagnosis of atopic dermatitis. To better reduced the recall bias caused by parental feedback, we designed this unified questionnaire, with clear definitions and objective standards for every measurement indicator. Second, we trained the investigator to fully understand the purpose and connotation of each question with the essential skills of collecting information. Third, we obtained the outcome information parallelly for both exposed and non-exposed populations via the same methodology to reduce the shortcomings of retrospective studies. In addition, the lack of detailed information on the parents was noticed, including a family history of allergies, and smoking history. Finally, given to the low rates of GBS colonization in this region, the smaller population receiving GBS-IAP may have influenced the outcome. Therefore, a prospective large-sample study will be established to collect the feces of newborns after IAP intervention and send for metagenome sequence. A longitudinal study will be conducted to investigate the impact of IAP on children to better understand the risks and benefits of the current GBS-IAP intervention strategies.

## Conclusion

GBS-IAP intervention for pregnant mothers significantly increased the incidence of AD in children aged 2 years in vaginal delivery.

## Supplementary Information

**Additional file 1: Supply Table 1.** Information, data sources and definitions of model covariates.

## Data Availability

The datasets generated and/or analyzed during the current study are not publicly available due to limitations of ethical approval involving the patient data and anonymity but are available from the corresponding author on a reasonable request.
