# Supplementary material for: A cohort study of intrapartum group B streptococcus prophylaxis on atopic dermatitis in 2-year-old children
Source: BMC Pediatr. 2022 Dec 2;22:693. doi: 10.1186/s12887-022-03758-5 (PMC9716662; doi:10.1186/s12887-022-03758-5)
Supplement: Supplementary file 1 — Additional file 1: Supply Table 1. Information, data sources and definitions of model covariates. [file 12887_2022_3758_MOESM1_ESM.docx]

Supply table 1. Information, data sources and definitions of model covariates.

| **Variable** | **Source** | **Definition** |
| --- | --- | --- |
| **Artificial feeding** | Labor room delivery summary | Infant is fed formula. |
| **Breast Feeding** | Labor room delivery summary | Exclusive breastfeeding within 6 months of birth was recorded as breastfeeding. |
| **Induced labor** | Labor room delivery summary | Induced labour is a medical treatment to start labour. |
| **Information on mother-child pairs** | Labor room delivery summary | One mother corresponds to one baby after removing confounding factors. |
| **Maternal pre-pregnacy BMI** | Labor room delivery summary | Prepregnancy BMI was calculated and categorized: underweight (BMI <18.5), normal weight (BMI 18.5–24.9), overweight (BMI 25.0–29.9), obese class I (BMI 30.0–34.9), and obese class II/III (BMI ≥35.0). |
| **Miscarriage** | Labor room delivery summary | Miscarriage is defined as a baby dies in the womb before 20 weeks of pregnancy. |
| **Mixed feeding** | Labor room delivery summary | Mixed feeding is when a baby is fed formula as well as breastmilk. |
| **Multipara** | Labor room delivery summary | more than one pregnancy resulting in viable offspring. |
| **Parity** | Labor room delivery summary | The number of times that she has given birth to a fetus with a gestational age of 24 weeks or more, regardless of whether the child was born alive or was stillborn. |
| **Primipara** | Labor room delivery summary | Only one pregnancy resulting in viable offspring. |
| **Weight of birth** | Labor room delivery summary | As reported, the body weight of a baby at its birth. |
| **GBS colonization** | Maternal medical record | GBS colonization was determined according to the screening results of prenatal GBS-PCR. A positive test indicates maternal GBS colonization. |
| **Gestational age** | Maternal medical record | Gestational age is a measure of the age of a pregnancy which is taken from the beginning of the woman's last menstrual period. |
| **IAP** | Maternal medical record | Those pregnant mothers with GBS positive received IAP at the onset of labor or at the time of rupture of membranes, whiles antibiotics are not required for those with cesarean section. Penicillin G or ampicillin, or cefazolin were being administered ≥4 hours before delivery. |
| **Infant diseases** | Maternal medical record | Newborns with major birth abnormalities or congenital developmental malformations that may lead to chronic disease. |
| **Maternal age** | Maternal medical record | The age of the mother at the time of delivery. |
| **Maternal intrapartum antibiotics** | Maternal medical record | The antibiotics assumed by pregnant women according to the medical records. |
| **Mother's allergy disease history** | Maternal medical record | The records of conditions caused by hypersensitivity of the immune system for the pregnant mother. |
| **Neuropsychiatric disorders** | Maternal medical record | https://www.nicklauschildrens.org/conditions/neuropsychiatric-disorders |
| **With IAP** | Maternal medical record | Those pregnant mothers with GBS positive received IAP treatment. |
| **Antibiotics use** **within 72 hours after birth** | Pediatric medical record | According to the medical records that whether the newborn has assumed antibiotics within 72 hours after birth. |
| **Keeping pets** | Questionnaire conducted in labor room | There are pets such as cats and dogs at home. |
| **Mother education level** | Questionnaire conducted in labor room | As reported. |
| **Mother's age** | Questionnaire conducted in labor room | As reported. |
| **Residence time** | Questionnaire conducted in labor room | Information was recorded according to the resident card. |
| **Smoking** | Questionnaire conducted in labor room | The mother was a smoker during pregnancy and after childbirth. |
| **Atopic dermatitis** | William’s criteria, diagnostic codes | The diagnostic of AD in infants was confirmed by > 3 criteria according to Williams standards (10), *i.e.* i) history of flexion involvement, including cubital fossa, popliteal fossa, anterior ankle, neck, including cheek rash in children under 10 years; ii) history of asthma or allergic rhinitis or a history of atopic disease in a first-degree relative in a child under 4 years of age; iii) history of dry skin all over the body; iv) flexor eczema, and cheek/forehead and extensor eczema in children under 4 years; v) onset before 2 years of age (for patients > 4 years of age). |
